# Corneal nerve loss as a surrogate marker for poor pial collaterals in patients with acute ischemic stroke

**DOI:** 10.1038/s41598-021-99131-0

**Published:** 2021-10-05

**Authors:** Adnan Khan, Ajay Menon, Naveed Akhtar, Saadat Kamran, Ahmad Muhammad, Georgios Ponirakis, Hoda Gad, Ioannis N. Petropoulos, Faisal Wadiwala, Blessy Babu, Adeeb M. Narangoli, Pablo G. Bermejo, Hanadi Al Hamad, Marwan Ramadan, Peter Woodruff, Mark Santos, Maher Saqqur, Ashfaq Shuaib, Rayaz A. Malik

**Affiliations:** 1 Weill Cornell Medicine-Qatar, Doha, Qatar; 2 Institute of Neuroscience, Hamad Medical Corporation, Doha, Qatar; 3 Department of Neurology, John Hunter Hospital, Newcastle, NSW Australia; 4 Geriatric, Rumailah Hospital, Hamad Medical Corporation, Doha, Qatar; 5 Psychiatry Hospital, Hamad Medical Corporation, Doha, Qatar; 6 Department of Neuroscience, School of Medicine, University of Sheffield, Sheffield, UK; 7 Trillium Hospital, University of Toronto at Mississauga, Mississauga, ON Canada; 8 Department of Medicine, University of Alberta, Alberta, Canada

**Keywords:** Biomarkers, Neurology

## Abstract

In patients with acute ischemic stroke, pial collaterals play a key role in limiting neurological disability by maintaining blood flow to ischemic penumbra. We hypothesized that patient with poor pial collaterals will have greater corneal nerve and endothelial cell abnormalities. In a cross-sectional study, 35 patients with acute ischemic stroke secondary to middle cerebral artery (MCA) occlusion with poor (n = 12) and moderate-good (n = 23) pial collaterals and 35 healthy controls underwent corneal confocal microscopy and quantification of corneal nerve and endothelial cell morphology. In patients with MCA stroke, corneal nerve fibre length (CNFL) (P < 0.001), corneal nerve fibre density (CNFD) (P = 0.025) and corneal nerve branch density (CNBD) (P = 0.002) were lower compared to controls. Age, BMI, cholesterol, triglycerides, HDL, LDL, systolic blood pressure, NIHSS and endothelial cell parameters did not differ but mRS was higher (p = 0.023) and CNFL (p = 0.026) and CNBD (p = 0.044) were lower in patients with poor compared to moderate-good collaterals. CNFL and CNBD distinguished subjects with poor from moderate-good pial collaterals with an AUC of 72% (95% CI 53–92%) and 71% (95% CI 53–90%), respectively. Corneal nerve loss is greater in patients with poor compared to moderate-good pial collaterals and may act as a surrogate marker for pial collateral status in patients with ischemic stroke.

## Introduction

Stroke is a major cause of disability and the second leading cause of death^1^. Ischemic stroke typically occurs following occlusion of a cerebral artery due to in situ thrombosis or an embolus from the heart or neck vessels^2^. Several studies have shown that the “leptomeningeal collateral circulation”, or “pial collateral status” determines the neurological outcome following acute ischemic stroke^3^. Pial collaterals are anastomotic connections located on the pial surface of the cortex that connect distal branches of the anterior, middle and posterior cerebral arteries, which permit blood flow from the territory of an unobstructed artery into the territory of an occluded artery^4^. Indeed, patients with poor pial collaterals sustain larger infarcts, respond poorly to reperfusion, have increased risk for and severity of intracerebral hemorrhage and suffer increased morbidity and mortality^3,5–7^, whereas those with good collaterals have better reperfusion, smaller infarcts and less hemorrhagic transformation^4^. A method to help to identify the pial collateral status in stroke patients may allow risk stratification and more aggressive or targeted interventions to reduce neurologic disability.

There is considerable variability in the pial collateral status amongst patients with acute ischemic stroke^8,9^. Along with genetics factors, several modifiable risk factors such as hypertension^10^, metabolic syndrome, hyperuricemia^11^, smoking^12^ and hyperglycemia^13^ are associated with poor collaterals. Animal studies have shown that “rarefaction of collaterals” is associated with multiple cardiovascular risk factors^14^. MR imaging has also shown that the presence of white matter hyperintensities is associated with a poor pial collateral circulation^15^ and disease severity is associated with poorer outcomes after stroke^16^.

Corneal confocal microscopy (CCM) is a rapid non-invasive ophthalmic imaging technique that has been used to demonstrate axonal loss in patients with impaired glucose tolerance^17^, diabetes^18,19^, and other peripheral neuropathies^20^. Our recent studies have also demonstrated a significant reduction in corneal nerves^21–23^ and abnormalities in corneal endothelial cells in patients with TIA^24^ and acute ischemic stroke^23^. We have also shown that corneal nerve loss is associated with age, HbA_1c_, lipids and blood pressure^19^ and the presence of white matter hyperintensities^25^ in patients with acute ischemic stroke.

The aim of this study was to assess if corneal confocal microscopy-based quantification of corneal nerve and endothelial cell abnormalities could act as surrogate markers for the pial collateral status in patients with acute ischemic stroke.

## Results

Thirty-five patients with acute MCA stroke were age-matched with thirty-five healthy controls (years) (51.54 ± 10.50 vs 52.82 ± 17.88, *p* = 0.717). The systolic blood pressure (mmHg) (144.71 ± 24.44 vs 132.31 ± 16.06, *p* = 0.014) was higher and HDL (mmol/l) 0.88 ± 0.21 vs 1.32 ± 0.37, *p* < 0.001) was lower in patients with stroke compared to controls.

### Clinical, metabolic and neurological disability according to pial collateral status

Patients with MCA stroke were classified into those with poor (n = 12) and moderate-good (n = 23) pial collaterals. Age (years) (52.42 ± 8.39 vs 51.09 ± 11.60, *p* = 0.797), gender (M/F) (21/02 vs 12/00, *p* = 0.293), BMI (kg/m^2^) (27.88 ± 3.40 vs 27.73 ± 4.39, *p* = 0.919), total cholesterol (mmol/l) (4.76 ± 1.26 vs 4.92 ± 0.97, *p* = 0.694), triglycerides (mmol/l) (1.59 ± 0.75 vs 1.68 ± 0.77, *p* = 0.632), LDL-cholesterol (mmol/l) (3.14 ± 1.00 vs 3.28 ± 0.89, *p* = 0.686), HDL-cholesterol (mmol/l) (0.91 ± 0.29 vs 0.87 ± 0.15, *p* = 0.626), systolic blood pressure (mmHg) (145.00 ± 20.32 vs 144.57 ± 26.76, *p* = 0.961) and HbA_1c_ (%) (7.29 ± 3.69 vs 6.02 ± 1.16, *p* = 0.294) did not differ significantly between patients with poor compared to moderate-good pial collaterals (Table 1).

**Table 1:** Demographic, metabolic, and clinical characteristics of healthy controls and participants with acute ischemic stroke with moderate-good and poor pial collaterals expressed as mean ± SD.

Parameters	All control (n = 35)	All stroke (n = 35)	P-value	Moderate-good collateral (n = 23)	Poor collateral (n = 12)	P-value
Age (years)	52.82 ± 17.88	51.54 ± 10.50	0.717	51.09 ± 11.60	52.42 ± 8.39	0.797
Gender (M/F)	**22/13**	**33/02**	< 0.001*	21/02	12/00	0.293
BMI (kg/m^2^)	27.89 ± 5.06	27.79 ± 4.01	0.926	27.73 ± 4.39	27.88 ± 3.40	0.919
Systolic blood pressure (mmHg)	**132.31 ± 16.06**	**144.71 ± 24.44**	0.014*	144.57 ± 26.76	145.00 ± 20.32	0.961
HbA_1c_ (%)	5.59 ± 0.42	6.45 ± 2.37	0.055	6.02 ± 1.16	7.29 ± 3.69	0.294
Total cholesterol (mmol/l)	4.95 ± 0.87	4.86 ± 1.07	0.715	4.92 ± 0.97	4.76 ± 1.26	0.694
Triglycerides (mmol/l)	1.60 ± 1.48	1.65 ± 0.75	0.887	1.68 ± 0.77	1.59 ± 0.75	0.632
LDL (mmol/l)	2.98 ± 0.72	3.23 ± 0.92	0.251	3.28 ± 0.89	3.14 ± 1.00	0.686
HDL (mmol/l)	**1.32 ± 0.37**	**0.88 ± 0.21**	< 0.001*	0.87 ± 0.15	0.91 ± 0.29	0.626
mRS at admission	NA	2.15 ± 1.48	NA	**1.68 ± 1.25**	**3.00 ± 1.54**	0.023*
mRS 90 days after discharge	NA	1.30 ± 1.46	NA	0.83 ± 1.04	2.22 ± 1.79	0.067
NIHSS at admission	NA	12.09 ± 6.30	NA	10.68 ± 5.96	14.67 ± 6.31	0.065
NIHSS 90 days after discharge	NA	5.23 ± 5.60	NA	3.76 ± 4.72	8.00 ± 6.32	0.120

*Statistically significant differences between groups.

The modified Rankin Scale (mRS) at admission (3.00 ± 1.54 vs 1.68 ± 1.25, *p* = 0.023) was significantly higher (44%) in patients with poor compared to moderate-good pial collaterals. Although not significant, the mRS at discharge (mRS: 2.22 ± 1.79 vs 0.83 ± 1.04, *p* = 0.067), and the National Institute of Health Stroke Scale (NIHSS) at admission (14.67 ± 6.31 vs 10.68 ± 5.96, *p* = 0.065) and at discharge (8.00 ± 6.32 vs 3.76 ± 4.72, *p* = 0.120) were 63%, 37% and 53% higher in patients with poor compared to moderate-good pial collaterals, respectively (Table 1).

### Corneal nerve and endothelial cell parameters in patients with acute ischemic stroke compared to controls

**Figure 1:**
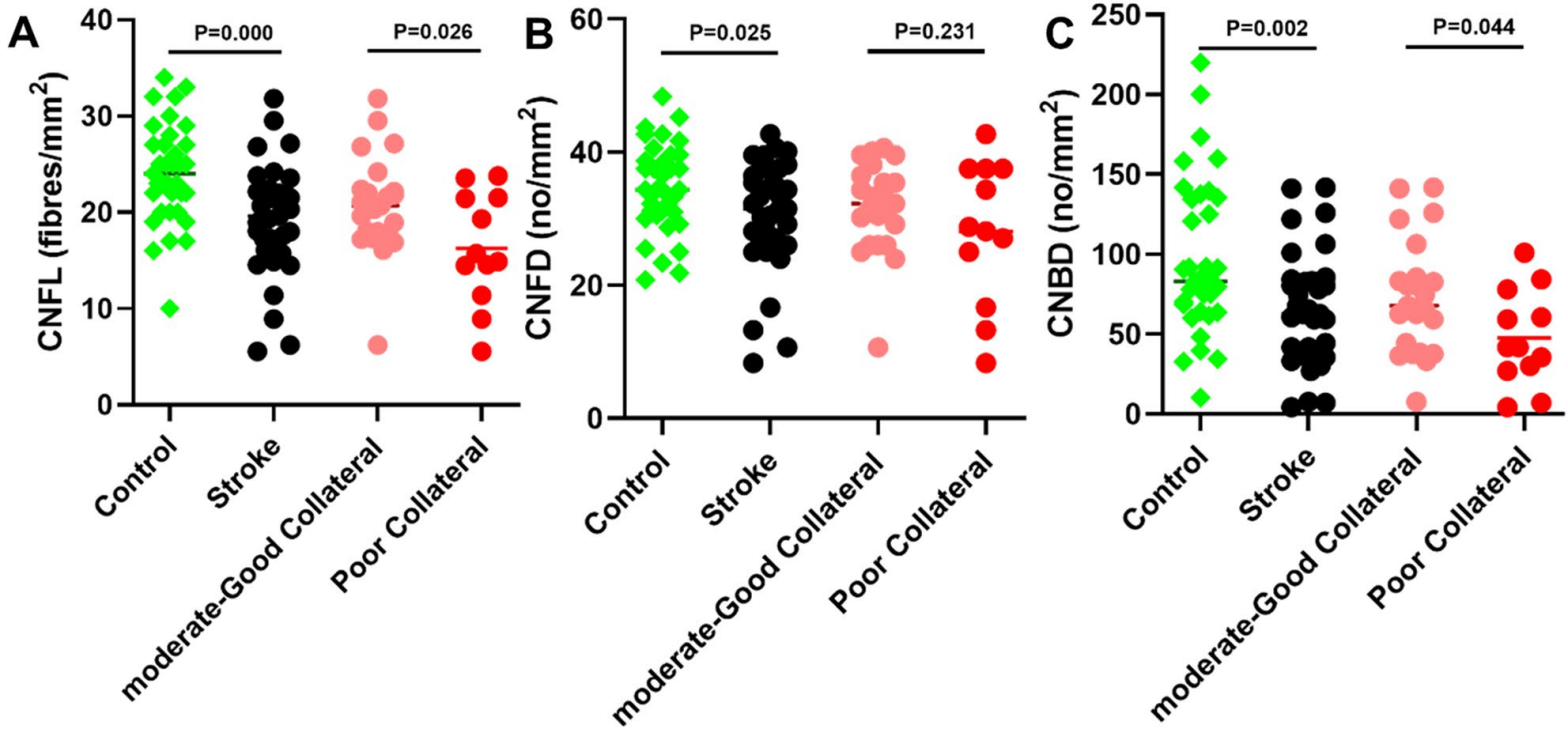
Dot plots of corneal nerve fiber parameters: (**A**) corneal nerve fiber length (CNFL), (**B**) corneal nerve fiber density (CNFD), (**C**) and corneal nerve branch density (CNBD), in controls, all stroke and stroke participants with poor compared to moderate-good collaterals.

**Figure 2:**
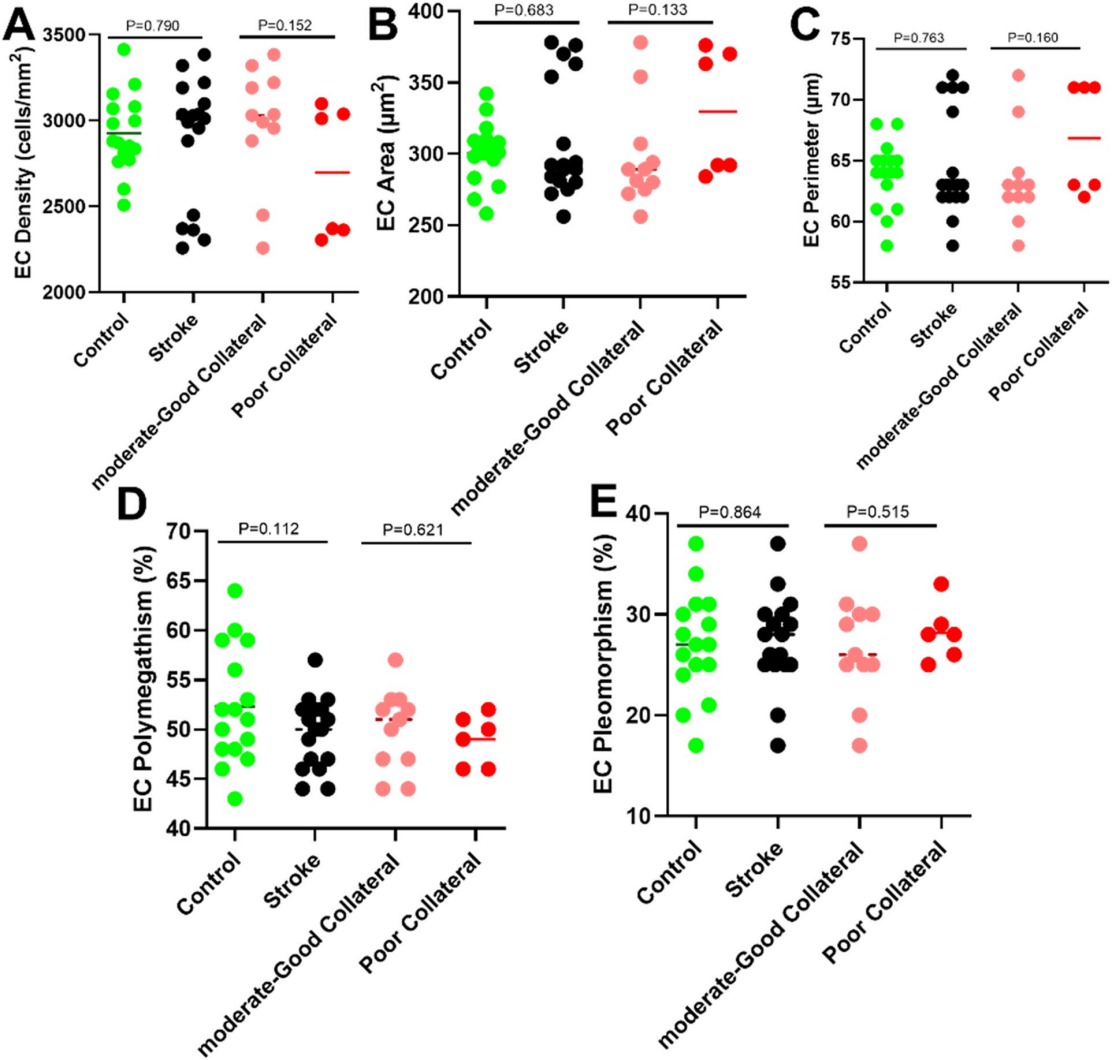
Dot plots of corneal endothelial cell (EC) parameters: (**A**) endothelial cell density, (**B**) endothelial cell area, (**C**) endothelial cell perimeter, (**D**) endothelial cell polymegathism (**E**) and endothelial cell pleomorphism in patients with poor compared to moderate-good collaterals.

CNFL (mm/mm^2^;19.22 ± 5.78 vs 24.03 ± 5.21, *p* < 0.001), CNFD (no/mm^2^; 30.45 ± 8.41 ± 34.62 ± 6.70, *p* = 0.025) and CNBD (no/mm^2^; 64.20 ± 35.70 vs 97.31 ± 48.21, *p* = 0.002) were 20%, 12% and 34% lower in patients with MCA stroke compared to controls (Fig. 1, Table 2). There was no significant difference in corneal endothelial cell density (ECD) (no./mm^2^) (2876.61 ± 374.58 vs 2924.95 ± 229.20, *p* = 0.790), endothelial cell area (ECA) (µm^2^) (308.91 ± 41.19 vs 300.41 ± 21.57, *p* = 0.683), endothelial cell perimeter (ECP) (µm) (64.64 ± 4.33 vs 63.75 ± 2.60, *p* = 0.763), endothelial cell polymegathism (%) (49.62 ± 3.61 vs 52.38 ± 5.87, *p* = 0.112) or pleomorphism (%) (27.25 ± 4.64 vs 26.95 ± 5.10, *p* = 0.864) between patients with stroke compared to controls (Fig. 2, Table 2).

**Table 2:** Corneal nerve and endothelial cell measures comparing controls to patients with acute ischemic stroke and between patients with moderate-good and poor collaterals expressed as mean ± SD.

Corneal nerve parameters	Control (n = 35)	All stroke (n = 35)	P-value	Moderate-good collateral (n = 23)	Poor collateral (n = 12)	P-value
**Corneal nerve parameters**						
CNFL, mm/mm^2^	**24.03 ± 5.21**	**19.22 ± 5.78**	< 0.001*	**20.76 ± 5.22**	**16.26 ± 5.84**	0.026*
CNFD, no/mm^2^	**34.62 ± 6.70**	**30.45 ± 8.41**	0.025*	31.70 ± 6.85	28.07 ± 10.74	0.231
CNBD, no/mm^2^	**97.31 ± 48.21**	**64.20 ± 35.70**	0.002*	**72.89 ± 35.89**	**47.53 ± 30.04**	0.044*

Endothelial cell parameters	Control (n = 16)	All stroke (n = 17)	P-value	Moderate-good collateral (n = 11)	Poor collateral (n = 6)	P-value
**Corneal endothelial cell parameters**						
ECD, cells/mm^2^	2924.95 ± 229.20	2876.61 ± 374.58	0.790	2974.11 ± 346.57	2697.86 ± 386.66	0.152
ECA, µm^2^	300.41 ± 21.57	308.91 ± 41.19	0.683	297.71 ± 36.6	329.44 ± 44.37	0.133
ECP, µm	63.75 ± 2.60	64.64 ± 4.33	0.763	63.54 ± 3.9	66.67 ± 4.68	0.160
EC Polymegathism, %	52.38 ± 5.87	49.62 ± 3.61	0.112	49.95 ± 4.14	49.00 ± 2.6	0.621
EC Pleomorphism, %	26.95 ± 5.10	27.25 ± 4.64	0.864	26.68 ± 5.43	28.28 ± 2.82	0.515

*Statistically significant differences between groups tested using t-test at p < 0.05 (data in bold).

### Corneal nerve and endothelial cell parameters in patients with poor compared to good pial collaterals

**Figure 3:**
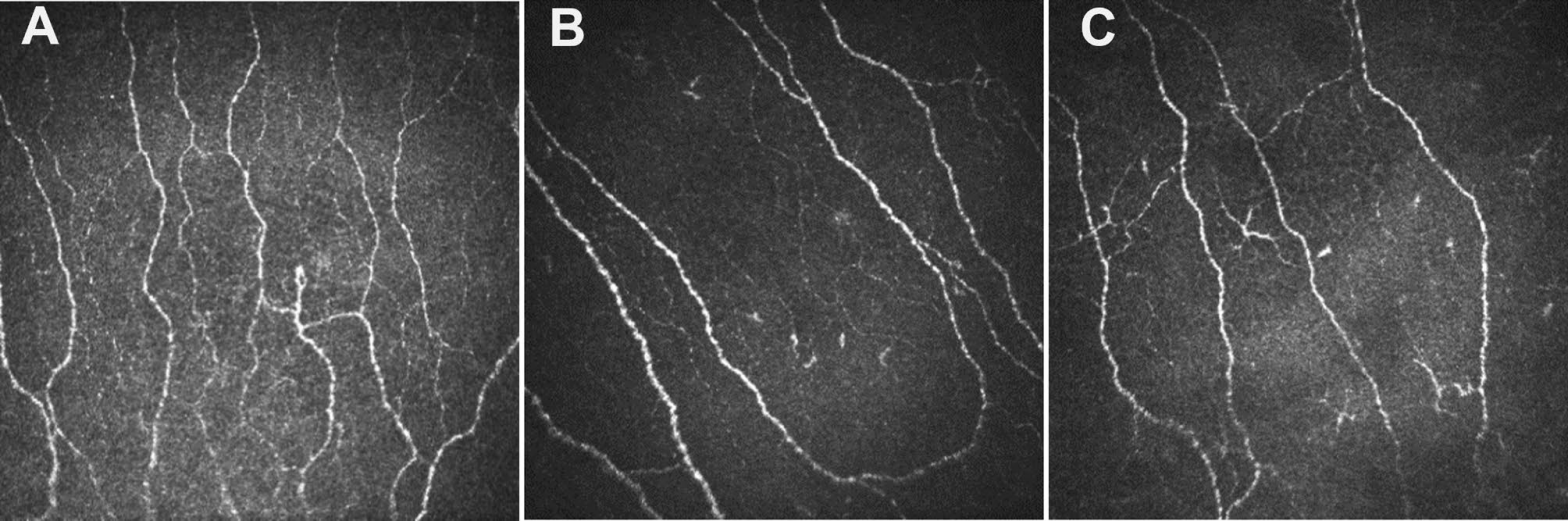
Corneal nerve morphology in a healthy control (**A**), patient with moderate-good pial collaterals (**B**) and patient with poor pial collaterals (**C**).

CNFL (mm/mm^2^) (16.26 ± 5.84 vs 20.76 ± 5.22, *p* = 0.026) and CNBD (no/mm^2^) (47.53 ± 30.04 vs 72.89 ± 35.89, *p* = 0.044) were 22% and 35% lower, with no difference in CNFD (*p* = 0.231) between patients with poor compared to moderate-good collaterals, respectively (Table 2, Fig. 3). There was no significant difference in ECD (no./mm^2^) (2697.86 ± 386.66 vs 2974.11 ± 346.57, *p* = 0.152), ECA (µm^2^) (329.44 ± 44.37 vs 297.71 ± 36.6, *p* = 0.133), ECP (µm) (66.67 ± 4.68 vs 63.54 ± 3.9, *p* = 0.160), endothelial cell polymegathism (%) (49.00 ± 2.6 vs 49.95 ± 4.14, *p* = 0.621) or pleomorphism (%) (28.28 ± 2.82 vs 26.68 ± 5.43, *p* = 0.515) between patients with poor compared to moderate-good collaterals (Fig. 2, Table 2).

### Diagnostic accuracy for distinguishing patients with poor from moderate-good collaterals

**Figure 4:**
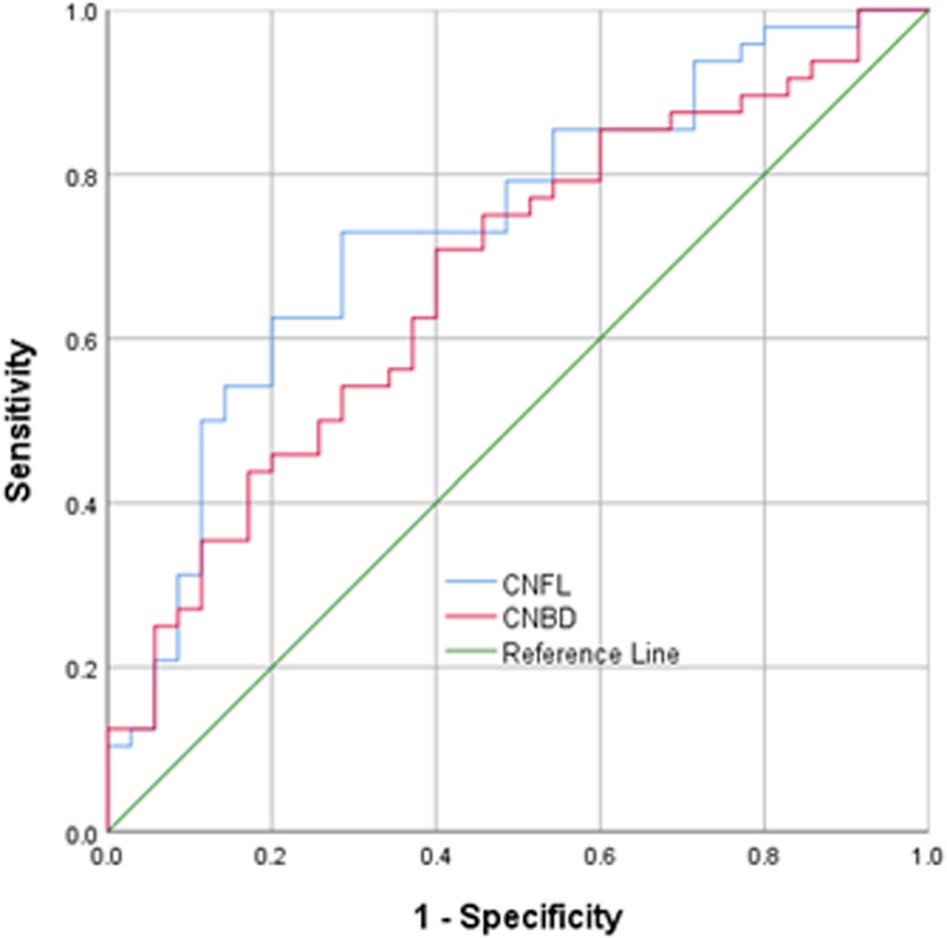
ROC analysis showing the area under the curve for corneal nerve fiber length and branch density for differentiating patients with poor from those with moderate-good pial collaterals.

Table 3 and Fig. 4 show the diagnostic accuracy of CCM measures for identifying patients with poor compared to moderate-good collaterals. CNFL and CNBD distinguished subjects with poor from good collaterals with 72% AUC (95% CI 53–92%) and 71% AUC (95% CI 53–90%), respectively. Using an abnormal cutoff of CNFL ≤ 16 the sensitivity and specificity were 96% and 58%, respectively, and using an abnormal cutoff of CNBD ≤ 62 the sensitivity and specificity were 65% and 75% according to the Youden index.

**Table 3:** Receiver operating characteristic (ROC) curve analysis for the diagnostic accuracy of corneal confocal microscopy for identifying patients with poor compared to moderate-good collaterals.

CCM parameters	AUC % (95% Cl)	P value	Cutoff point	Sensitivity (%)	Specificity (%)
CNFL, mm/mm^2^	72 (53–92)	= 0.034	≤ 16	96	58
CNBD, no./mm^2^	71 (53–90)	= 0.040	≤ 62	65	75

## Discussion

In this study, we show evidence of corneal nerve loss in patients with acute MCA stroke which agrees with our recent studies in patients with TIA^24^, acute^21,23^ and recurrent stroke^22^. Moreover, we show that corneal nerve loss was greater and there was good diagnostic accuracy for differentiating patients with poor compared to moderate-good pial collaterals, despite comparable blood pressure, lipids and HbA_1c_. We have previously shown corneal endothelial cell abnormalities in patients with TIA and stroke^23^, however, the current study showed no differences between patients with MCA stroke and controls or between patients with good compared to poor pial collaterals.

Although the pial collateral status plays a major role in determining neurological outcomes in acute ischemic stroke it can only be ascertained following the occurrence of large cerebral artery occlusion. White matter hyperintensities (WMH) predict poor stroke outcomes 90 days^26–28^ after thrombectomy and the presence of WMH was associated with greater cerebrovascular dysfunction in patients with large vessel occlusion^15^, whilst those without WMH had more favorable outcomes in the ASPECT score^28^. WMH are also associated with endothelial dysfunction^29^ and poor pial collateral circulation^15^. Indeed, many of the risk factors and comorbidities associated with WMHs have also been associated with poor pial collateral status. WMH increase with age^30^, hypertension^31,32^ and diabetes^33,34^ and may improve with antiplatelet therapy^35^ and improved management of hypertension^31,32^ and diabetes^33,34^. Of relevance to the current study, these same risk factors have been related to corneal nerve degeneration^36^ and indeed improvement in blood pressure, lipids and glycemic control is associated with corneal nerve regeneration^37,38^. Moreover, recently we showed that CCM may act as a surrogate imaging marker for the presence and severity of WMHs in patients with acute ischemic stroke^25^.

CCM has emerged as a powerful non-invasive ophthalmic imaging endpoint to identify corneal nerve loss as a surrogate marker for neurodegeneration in patients with multiple sclerosis^39^, Parkinson’s disease^40^, dementia^41^and patients with acute ischemic stroke^21^ and recurrent stroke^22^. We now show that CCM identifies greater corneal nerve loss in patients with poor compared to moderate/good pial collaterals. This ophthalmic imaging method may therefore act as a surrogate marker for poor pial collaterals and allow the identification of patients who require greater risk factor reduction and more urgent reperfusion after ischemic stroke. Indeed, in the present study patients with poor pial collaterals had a higher mRS and a previous study showed a larger infarct volume and higher mRS and NIHSS in patients with worse pial collateral scores^42^.

Limitations of the current study include the moderate sample size and the assessment of only patients with moderate disability who could undergo CCM. However, our study broadens the clinical utility of CCM in patients with neurodegenerative disease and patients with ischemic stroke. These data warrant larger studies utilising CCM in patients with or who are at risk of ischemic stroke.

## Materials and methods

Thirty-five patients with middle cerebral artery occlusion and 35 age-matched healthy control participants attending the geriatric department at Hamad Medical Corporation were recruited from 2016 to 2018 in Doha, Qatar. In this cross-sectional study, exclusion criteria for participants with ischemic stroke were stroke due to a non-vascular disorder, or intracerebral hemorrhage, a known history of ocular trauma or surgery, high refractive error, and glaucoma. Healthy controls participants without known ocular or systemic co-morbidities including diabetes mellitus were also recruited.

Acute ischemic stroke was confirmed clinically and radiologically using American Heart Association (AHA) criteria^43^. The pial collateral status was established using multi-modal/dynamic CTA according to the criteria of Tan et al.^44^ by an investigator who was blinded to the patient’s corneal morphology status. The ordinal collateral score ranges from 0 to 3: 0 = absent collateral supply to the occluded MCA territory, 1 = collateral supply filling ≤ 50% but > 0% of the occluded MCA territory, 2 = collateral supply filling > 50% but < 100% of the occluded MCA territory and 3 = 100% collateral supply of the occluded MCA territory. Patients with pial collateral vessels filling equal to or less than 50% of the occluded middle cerebral artery territory were defined as those with ‘poor collaterals’ whereas patients with collateral vessels filling more than 50% of the occluded middle cerebral artery territory were defined as those with ‘moderate- good collaterals’.

Clinical and demographic data along with blood pressure, HbA_1c_ and lipid profile were obtained at admission. The National Institutes of Health Stroke Scale (NIHSS)^45^ and modified Rankin Scale (mRS)^46^ was obtained for all patients at admission and at discharge from hospital. This study adhered to the tenets of the declaration of Helsinki and was approved by the Institutional Review Board of Weill Cornell Medicine (15-00021) and Hamad Medical Corporation (15304/15). Informed, written consent was obtained from all patients/guardians before participation in the study.

### Corneal confocal microscopy

All patients underwent CCM (Heidelberg Retinal Tomograph III Rostock Cornea Module; Heidelberg Engineering GmbH, Heidelberg, Germany). CCM uses a 670 nm wavelength helium neon diode laser, which is a class I laser and therefore does not pose any ocular safety hazard. A × 63 objective lens with a numeric aperture of 0.9 and a working distance, relative to the applanating cap (TomoCap; Heidelberg Engineering GmbH) of 0.0 to 3.0 mm, is used. The size of each 2-dimensional image produced is 384 × 384 pixels with a 15° × 15° field of view and 10 μm/pixel transverse optical resolutions. To perform the CCM examination, local anesthetic (0.4% benoxinate hydrochloride; Chauvin Pharmaceuticals, Chefaro, United Kingdom) was used to anesthetize both eyes, and Viscotears (Carbomer 980, 0.2%, Novartis, United Kingdom) was used as the coupling agent between the cornea and the cap. Patients were asked to fixate on an outer fixation light throughout the CCM scan and a CCD camera was used to correctly position the cap onto the cornea^20^. The examination took approximately 10 min for both eyes. The examiners captured images of the central sub-basal nerve plexus using the section mode. On the basis of depth, contrast, focus, and position, 6 images per patient were selected^47^.

All CCM images were manually analysed using validated, purpose-written software by an investigator blinded to the collateral status of the participants. Corneal nerve fiber density (CNFD: total number of major nerves/mm^2^), corneal nerve branch density (CNBD: number of branches emanating from major nerve trunks/mm^2^), corneal nerve fiber length (CNFL: total length of all nerve fibers and branches mm/mm^2^) and inferior whorl length (IWL: total length of all nerve fibers in the inferior whorl area mm/mm^2^) were analyzed using CCMetrics (M. A. Dabbah, ISBE, University of Manchester, Manchester, United Kingdom)^18^. Corneal endothelial cell images were analyzed using the Corneal Endothelium Analysis System (CEAS), an automated image analysis system^48^. Endothelial cell density (ECD, cells/mm^2^), endothelial cell area (ECA, µm^2^), endothelial cell perimeter (ECP, µm), endothelial cell polymegathism (%) and endothelial cell pleomorphism (%) were quantified. Polymegathism was defined as the standard deviation of the cell area divided by the mean cell area, while pleomorphism was defined as the hexagonality coefficient. Adequate corneal endothelial cells images were available in seventeen patients with poor (n = 6) and moderate-good (n = 11) collaterals and sixteen healthy controls.

### Statistical analysis

All statistical analyses were performed using IBM SPSS Statistics software Version 25. Normality of the data was assessed using the Shapiro–Wilk test and by visual inspection of the histogram and a normal Q-Q plot. Data are expressed as mean ± standard deviation (SD). Mann Whitney test (for non-normally distributed variables) and t-test (for normally distributed variables) were performed to find the differences between two groups. Receiver operating characteristic (ROC) curve analysis was performed for corneal nerve parameters to identify patients with poor compared to moderate-good collateral status.

### Ethics approval

This study adhered to the tenets of the declaration of Helsinki and was approved by the Institutional Review Board of Weill Cornell Medicine (15-00021) and Hamad General Hospital (15304/15).

### Consent to participate

Informed, written consent was obtained from all patients/guardians before participation in the study.

### Consent for publication

Written consent was obtained from all patients/guardians for publications.

## Data Availability

The datasets generated during and/or analyzed during the current study are available from the corresponding author on reasonable request.
